# Advances in the multidisciplinary surgical approach to primary spinal sarcomas: insights from a retrospective case series on outcomes and survival

**DOI:** 10.1007/s00701-024-06199-4

**Published:** 2024-08-06

**Authors:** Pavlina Lenga, Philip Dao Trong, Helena Kleineidam, Andreas W. Unterberg, Sandro M. Krieg, Basem Ishak

**Affiliations:** 1 Department of Neurosurgery, Heidelberg University Hospital, Im Neuenheimer Feld 400, 69120 Heidelberg, Germany; 2 Medical Faculty of Heidelberg University, Heidelberg, Germany

**Keywords:** Spinal sarcoma, En-bloc resection, Complications, Morbidity

## Abstract

**Introduction:**

The management of spinal sarcomas is complex, given their widespread involvement and high recurrence rates. Despite consensus on the need for a multidisciplinary approach with surgery at its core, there is a lack of definitive guidelines for clinical decision-making. This study examines a case series of primary spinal sarcomas, focusing on the surgical strategies, clinical results, and survival data to inform and guide therapeutic practices.

**Methods:**

We conducted a retrospective analysis of patients who underwent surgical resection for primary spinal sarcomas between 2005 and 2022. The study focused on gathering data on patient demographics, surgical details, postoperative complications, overall hospital stay, and mortality within 90 days post-surgery.

**Results:**

The study included 14 patients with a primary diagnosis of spinal sarcoma, with an average age of 48.6 ± 12.6 years. Chondrosarcoma emerged as the most common tumor type, representing 57.1% of cases, followed by Ewing sarcoma at 35.7%, and synovial sarcoma at 7.1%. Patients with chondrosarcoma were treated with en-bloc resection, while the patient with synovial sarcoma underwent intra-lesional excision and those with Ewing sarcoma received decompression and tumor debulking. Postoperative assessments revealed significant improvements in neurological conditions. Notably, functional status as measured by the Karnofski Performance Index (KPI), improved substantially post-surgery (from 61.4 to 80.0%) The mean follow-up was 34.9 ± 9.2 months. During this time period one patient experienced fatal bleeding after en-bloc resection complications involving the vena cava. None of the patient needed further surgery.

**Conclusions:**

Our 16-year study offers vital insights into managing primary spinal sarcomas, showcasing the effectiveness of surgical intervention, particularly en-bloc resection. Despite their rarity and complexity, our multidisciplinary treatment approach yields improved outcomes and highlights the potential for refined surgical strategies to become standardized care in this challenging domain.

## Introduction

Primary spinal column tumors are rare, with over 90% representing metastases from solid tumors [24]. These tumors may originate from various non-epithelial tissues such as bone, cartilage, fat, muscles, and blood vessels. Notably, they account for less than 5% of all bone neoplasms and under 0.2% of all cancer types [10]. These tumors frequently affect multiple levels of the spinal cord, the paraspinal muscles, and the epidural space. Consequently, many patients experience progressively worsening pain and neurological decline. Their surgical management is complex due to the proximity and invasion of vital structures. Regardless of the sarcoma type, existing literature advocates for a multidisciplinary approach, with surgical resection as the primary treatment [9, 12, 13, 20, 21, 23]. Surgical resection is crucial as it may alleviate pain, maintain or enhance function, and significantly improve survival rates while reducing the chance of local recurrence.

While en-bloc resection with negative margins has been established as the primary modality for local tumor control and long-term survival in extraspinal [8, 22, 24, 29], its application in spinal sarcomas remains underexplored. The complexity of such procedures, the consequent need for significant reconstruction, and the associated increase in morbidity and mortality [31] are notable concerns. Moreover, the proximity to vital vascular structures and spinal roots often precludes the feasibility of complete resections [26] shifting the treatment goal towards preserving neurological function through decompression, followed by adjunctive chemotherapy and radiotherapy. The existing body of knowledge, largely derived from retrospective case series and singular case reports, falls short of providing comprehensive treatment guidelines for spinal sarcomas. This evidentiary gap hinders the development of standardized treatment protocols.

The urgent need to find novel treatment methods for spinal sarcomas is evident, demanding a deeper insight into the various mechanisms underlying these tumors' etiologies. Our study aims to present a case series of patients with primary spinal sarcomas, outlining surgical strategies, clinical outcomes, and survival rates.

## Methods

### Study design and inclusion criteria

This retrospective study analyzed clinical and imaging data spanning 16 years, from September 2005 to December 2023, obtained from a single-center database. The research, classified as a non-interventional study, received approval from the local ethics committee of our institution (approval number S518/2023) and adhered to the principles outlined in the Declaration of Helsinki. Informed consent was obtained from the parents or legal guardians of the patients. Participants included in the study were individuals aged 18 years or older, diagnosed with primary spinal sarcoma. Patients were excluded if they were under 18, had coexisting intracranial or cervical conditions, secondary spinal tumors, or incomplete data.

**Fig. 1:**
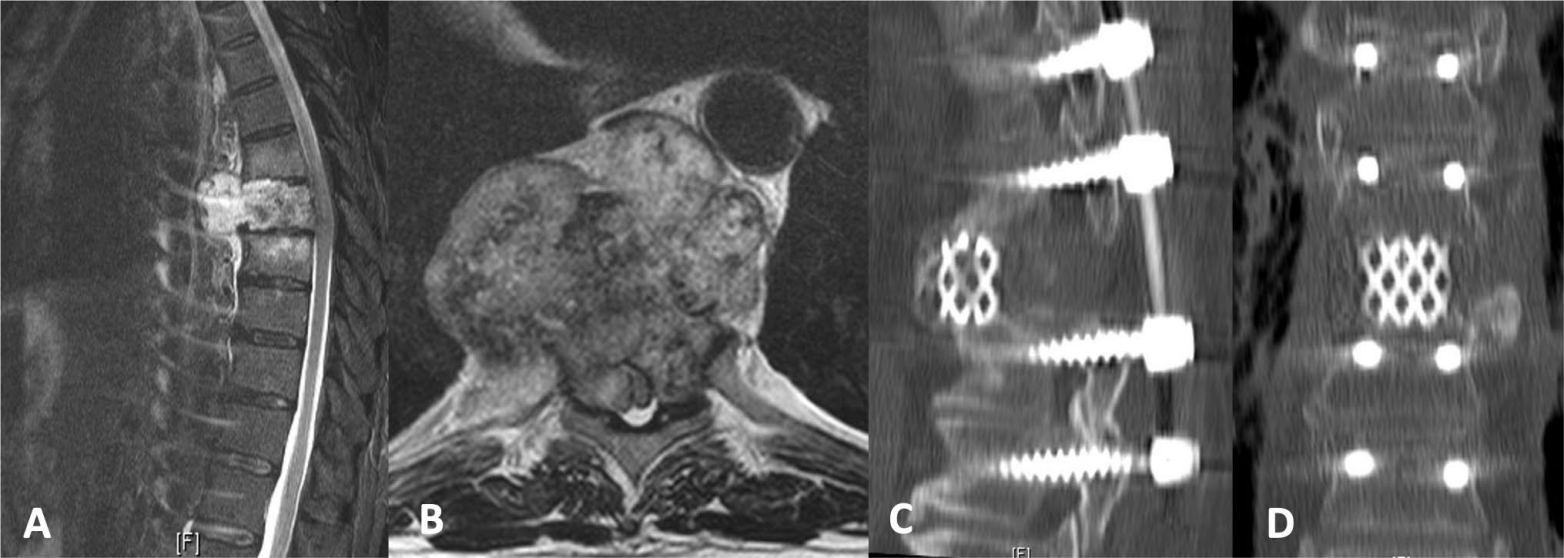
Preoperative and Postoperative Imaging of Spinal Sarcoma in a 69-Year-old male patient. (**A**) Sagittal T2-weighted MRI showing a hyperintense tumor mass extending from Th8, causing marked spinal cord compression. (**B**) Axial T2-weighted MRI at the level of Th8 revealing tumor invasion into the spinal canal and erosion of the right costo-transverse joint, indicative of aggressive pathology. (**C**) Postoperative sagittal CT scan illustrating the extent of vertebrectomy at T11 with the placement of spinal instrumentation. (**D**) Postoperative coronal CT scan demonstrating successful alignment and stabilization from T6 to L1 with pedicle screws and rod constructs, and evidence of vertebrectomy at T11

The study involved a comprehensive review of patient demographics, comorbidities, American Society of Anesthesiologists scores, duration of surgery, number of spinal levels treated, and various perioperative and postoperative outcomes. These outcomes included length of hospital stay, time in the intensive care unit, instances of readmission, need for reoperation, and mortality rates. Pre-treatment neurological condition was assessed using the Motor Score (MS) of the American Spinal Injury Association impairment grading system (MS = 0, no muscle strength; MS = 100, healthy). Post-treatment MS data were obtained from the last documented clinical encounter. The Karnofsky Performance Index (KPI) was used to assess changes in a patient’s condition, attributing grades to their ability to perform certain tasks according to the following scores: 100, normal state, no complaints; 70, inability to perform daily life activities; 50, requires significant assistance; 40, disability; 30, mandatory hospitalization; and 0, deceased [15]. The extent of spinal injury was evaluated using the Frankel Grade (FG): A, complete motor and sensory loss below the level of spinal cord dysfunction; B, sensation spared, but no motor activity; C, non-functional motor preservation; D, useful motor preservation; and E, normal motor function [11]. Routine clinical and radiological evaluations, including MRI scans of the spinal cord, were conducted prior to discharge and at the three-month postoperative mark to monitor for tumor recurrence (Fig. 1).

### Decision making

A patient exhibiting acute neurological deterioration was promptly subjected to decompression surgery to address the rapid onset of symptoms and prevent further neurological compromise. Decisions for such urgent surgeries were made swiftly by a core team comprising a neurosurgeon, a medical oncologist, and a radiologist, who collectively assessed the clinical and imaging data available at the time. During this procedure, a tissue sample was carefully extracted and submitted to the pathology department for comprehensive histological examination. This analysis is crucial for confirming the diagnosis and understanding the nature of the pathology involved. Following the surgical intervention and initial pathological assessment, the case was meticulously reviewed by an interdisciplinary tumor board. This board comprises specialists from oncology, neurosurgery, radiology, and pathology, who collaboratively discussed the patient's treatment options based on the latest clinical findings and histological data. The tumor board's insights are vital for devising a personalized treatment plan that optimally addresses the patient's specific condition. For elective cases, a planned biopsy was performed prior to any surgical intervention. This approach allows for a precise diagnosis and facilitates detailed planning of the surgical strategy in elective cases. It ensures that all therapeutic decisions are based on robust histological evidence, thereby enhancing treatment efficacy and patient safety.

In terms of surgical technique, various factors such as surgeon preferences, tumor location, size, and aggressiveness influenced the choice of procedure. Oncological considerations included the comparison of surgical options against radiation therapy and chemotherapy, the feasibility of complete resection following neoadjuvant therapy, and symptom response to steroids. Decisions regarding the surgical approach (en bloc resection, intralesional resection, or laminectomy with tumor debulking) were made during interdisciplinary conferences, focusing on the overall prognosis of the disease. Adjuvant chemotherapy and radiation therapy have been administered as salvage therapies, predominantly in patients after incomplete resection or in the palliative phase at the discretion of the treating physician.

### Statistical analysis

Categorical variables are presented as numbers and percentages. Continuous variables are presented as means ± standard deviations and the Shapiro–Wilk test was used to verify whether their distribution was normal. Baseline characteristics, duration of surgery, number of treated spinal levels, perioperative and postoperative complications, length of stay (LOS), ICU stay, readmissions, reoperations, and mortality were reported.

## Results

Over a period of 16 years, this study encompassed 14 patients diagnosed with primary spinal sarcoma. The average age of the patients was 48.6 ± 12.6 years. Notably, five patients experienced acute neurological deterioration. Within this subgroup, two patients were assessed with a Frankel Grade A motor deficit, and another two presented with Frankel Grade B motor deficits. Analysis of tumor locations indicated a higher incidence in the thoracic and thoracolumbar regions of the spine, with prevalence rates of 57.1% and 28.6%, respectively. Chondrosarcoma was identified as the most frequent type of tumor, accounting for 57.1% of the cases (8 out of 14). A comprehensive overview of the patient characteristics is presented in Table 1.

**Table 1:** Baseline characteristics

Characteristic	Value
Number of patients	14
Age, months (mean, SD,range)	48.6 (12.6;18–69)
Sex (n, %)	
Male	9 (64.3)
Female	5 (35.7)
ASA class (mean, SD)	2.5 (0.6)
Symptoms	
MS Score (mean, SD)	77.5 (16.8)
Frankel Grade (n, %)	
A	2 (14.3)
B	2 (14.3)
C	5 (35.7)
D	3 (21.4)
E	2 (14.3)
KPI (mean, SD)	61.4 (22.8)
Level of Spinal Cord Compression (n, %)	
Cervical	1 (7.1)
Thoracic	8 (57.1)
Thoracolumbar	4 (28.6)
Lumbar	1 (7.1)
Tumor entity (n, %)	
Chondrosarcoma	8 (57.1)
Synovialissarcoma	1 (7.1)
Ewing sarcoma	5 (35.7)

^*^ASA, American Society of Anesthesiologists; KPI, Karnofsky Performance Index; MS, motor score of the American Spinal Injury Association grading system; SD, standard deviation

### Surgical characteristics and clinical scores

As indicated in Table 2, the average duration of surgery was 9.5 h with an SD 2.5 h, with a range from 80 to 750 min. Patients with chondrosarcoma underwent en-bloc resection. In contrast, one patient with synovial sarcoma received intra-lesional excision, while those with Ewing sarcoma underwent decompression and tumor debulking. The average number of decompressed spinal levels was 2.3 ± 1.4. The mean stay in the Intensive Care Unit (ICU) was less than one day, and the overall hospital stay was 10.5 ± 3.8 days. During hospitalization, one patient succumbed to massive bleeding following en-bloc resection, which led to injury of the vena cava. Another patient required re-admission due to tumor progression2 months after initial surgery, but no additional surgeries were performed during the follow-up. Neurological and functional scores, including MS, FG, and KPI, showed significant improvement post-surgery as detailed in Table 2 and further analyzed in Table 3. The mean follow-up duration was 34.9 ± 9.2 months, and there was no need for additional surgery for secondary instability.

**Table 2:** Peri- and postoperative surgical characteristics and clinical course

Characteristic	All (n = 14)
Surgical duration	9.5 h (80;750 min)
Surgical approach	
Decompression	5 (35.7)
Intralesional excision	1 (7.1)
Decompression + ventrodorsal instrumentation (en bloc resection)	8 (57.1)
Number of levels decompressed	2.3 (1.4)
Hospital stay, days	10.5 (3.8)
ICU stay, days	0.6 (1.6)
Mortality	
In-hospital (n, %)	1 (7.1)
90-day (n, %)	1 (7.1)
30-day readmission (n, %)	1 (7.1)
MS after surgery (mean, SD)	82.5 (12.6)
Postoperative Frankel Grade (n, %)	
A	0 (0.0)
B	1 (7.1)
C	5 (35.7)
D	4 (28.6)
E	4 (28.6)
Postoperative KPI (mean, SD)	80.0 (11.5)
Chemotherapy after surgery (n, %)	5 (35.7)
Radiation after surgery (n, %)	9 (64.3)

The values are indicated as mean (SD) unless otherwise indicated

ICU, intensive care unit; MS, motor score of the American Spinal Injury Association grading system

**Table 3:** Occurrence of adverse

Event	All (n = 14)
Deep wound infection	1 (7.1)
Pneumonia	1 (7.1)
Urinary tract infection	1 (7.1)
Revision surgery	
Epidural hematoma	2 (14.2)
Dural leak	1 (7.1)

## Complications

The most common complications included deep wound infection, urinary tract infection, and pneumonia, each occurring at a prevalence of 7.1%. Three patients required revision surgery for complications such as epidural bleeding and dural leakage following en-bloc resection. Table 4 presents detailed data on postoperative complications.

## Discussion

The primary goals of surgical procedures are pain relief, preservation or improvement of neurological functions, and effective local tumor control. In our research, we analyzed 14 cases of primary spinal sarcomas addressed through surgical means. Each patient presented with acute pain or a new onset of neurological deficits, necessitating urgent surgical intervention. Our study reveals notable improvements in neurological status and disability scores postoperatively. As for the surgical approaches, patients with chondrosarcoma were treated with en-bloc resection, demonstrating zero recurrence rates, whereas patients with Ewing sarcoma underwent surgery followed by adjunct radiation and chemotherapy, effectively reducing their recurrence. A critical observation was the unfortunate demise of a patient due to extensive injury to the vena cava during the surgical procedure.

In the realm of spinal sarcoma treatment, surgical intervention has consistently been a subject of extensive research. A notable study by Bilsky et al. [4] involving 59 patients with spinal sarcoma provided critical insights into the outcomes of surgical treatment. They reported that patients with mild neurological deficits pre-surgery (categorized as ASIA C or D) predominantly remained stable postoperatively [4]. A significant finding from this study was that 13% of patients initially assessed as ASIA B experienced substantial improvement post-surgery, advancing to an ASIA D status. This study was pivotal in demonstrating that surgery could not only halt the progression of neurological deficits but also potentially reverse them. 95% of the patients in this cohort regained the ability to walk post-surgery, a key indicator of the success of surgical interventions in spinal sarcomas. Further expanding on this topic, Rao et al. [23] presented a comprehensive analysis of 110 spinal sarcoma surgeries [23]. Their study provided a broader perspective on the neurological outcomes post-surgery. They categorized 18 patients (16%) as having severe neurological deficits preoperatively (ASIA grades A to C), while the majority, 92 patients (84%), were assessed with milder impairments (ASIA grades D or E). In their follow-up, Rao et al. found that postoperative neurological statuses remained unchanged in 83% of the patients, improved in 14%, and, notably, worsened in 3%. While surgery itself predominantly stabilizes or improves neurological function, a small risk of deterioration has still to be taken into account. In a more specialized study focusing on synovial spinal sarcomas, Yang et al. [30] reported significant improvements in pain management and neurological function following surgery [30]. Their research is particularly noteworthy for the absence of severe postoperative neurological deficits among the patients, indicating the potential effectiveness of surgical interventions in this specific subtype of spinal sarcoma. In line with these studies, our current research also shows significant postoperative improvements. Notably, the average KPS in our study group was 80.0, indicating a high level of functional independence in daily life post-surgery. This improvement in motor function and overall quality of life provides further evidence supporting the role of surgery in managing spinal sarcomas. Our findings suggest that surgery is not just a treatment option but a key therapeutic strategy for these complex spinal tumors. It offers patients a chance not only to regain ambulatory status and manage pain but also to significantly improve their overall quality of life.

Pediatric primary sarcomas of the spine, such as Ewing's sarcoma, which represents a relatively uncommon subgroup, constituting 3.5% to 9.8% of all Ewing's cases [14], demonstrate the need for specialized treatment protocols. These tumors are particularly challenging due to their rarity, with only 65 cases noted across all age groups, and merely ten cases within the pediatric population [25, 27]. The main therapeutic interventions for spinal neuroectodermal tumors in children typically focus on surgical approaches aimed at preserving function, alleviating pain, removing the lesion, managing recurrence, and potentially prolonging survival [9]. However, most guidelines are based on limited evidence from case reports, case series, or retrospective studies, all of which highlight the urgent need for more robust data [18, 19]. Aggressive treatments necessary for these cases can significantly impact the long-term quality of life of pediatric patients, underscoring the need for refined protocols that balance curative intent with long-term well-being [5]. In contrast, the management strategies for adults, particularly when comparing younger adults around 18 years old with older adults over 60, significantly diverge. Younger adults often undergo aggressive surgical and adjuvant therapies aimed at curing the disease [32]. For older adults, however, the emphasis shifts towards preserving function and quality of life, guiding less aggressive interventions that focus on maintaining independence and minimizing treatment-related morbidity [16]. The differences in therapeutic goals across these age groups indicate a clear necessity for large, multicenter registries. Such registries would enable the collection of comprehensive data on spinal sarcomas, particularly rare pediatric cases. They are crucial for advancing our understanding of the disease and enhancing treatment strategies across diverse patient demographics. This improved data collection is essential not only for refining current protocols but also for ensuring that all patients receive care that is both effective and considerate of their long-term health and quality of life.

In recent years, the treatment paradigm for Ewing sarcoma, particularly in pediatric and adolescent patients, has evolved significantly. Neoadjuvant chemotherapy is now recognized as a cornerstone in the management of this aggressive tumor, aiming to reduce tumor size, facilitate surgical resection, and address microscopic metastatic disease that may not be detectable at diagnosis. Our study includes a pertinent case of an 18-year-old patient who underwent neoadjuvant chemotherapy followed by surgical resection. This case exemplifies the current clinical approach where neoadjuvant therapy is implemented to enhance the feasibility and efficacy of subsequent surgical interventions, such as en bloc resection. This approach aligns with the European Society for Medical Oncology (ESMO) guidelines, which recommend a combination of drugs including vincristine, doxorubicin, and cyclophosphamide, supplemented by ifosfamide and etoposide, as part of the neoadjuvant chemotherapy regimen (Bisogno et al., 2012). Following the chemotherapy, en bloc resection was performed, aiming to remove the tumor in one contiguous piece, thus minimizing the risk of local recurrence. This surgical technique, particularly advocated in cases where the tumor is localized and a complete resection is achievable, has been supported by studies such as those by Ladenstein et al. (2010), which demonstrate improved prognosis with complete surgical excision following effective chemotherapeutic shrinkage of tumors. The integration of neoadjuvant chemotherapy with en bloc resection has been shown to significantly improve survival rates, especially in younger patients. A retrospective analysis by Bacci et al. (2008) on pediatric patients with Ewing sarcoma reported a 5-year survival rate increase from 55 to 72% with the use of preoperative chemotherapy followed by surgical resection of the tumor [1].

In the surgical management of spinal tumors, the choice of technique is critically influenced by various factors, including the tumor's histology, surgical feasibility, lesion accessibility, and the patient's overall health condition. A significant contribution to this field comes from Boriani et al., who conducted a retrospective study on 22 chondrosarcoma cases [6].. Their findings suggest that en bloc resection is more effective in extending survival and controlling local tumor recurrence compared to intralesional resection. Specifically, they reported a recurrence rate of 21% and an overall survival rate of 90% (with an average follow-up of 97 months) in patients who underwent en bloc excisions. In contrast, patients who had intralesional resections experienced a 100% recurrence rate and only a 20% overall survival rate (average follow-up of 61 months) Boriani et al. [6]. This finding is echoed by Rao et al. (2008), who noted a local recurrence rate of 65% in patients undergoing intralesional excision, compared to just 20% in those who had en bloc resections (Rao et al., 2008). In our study, more than half of the cases involved en bloc tumor resection. Two patients with Ewing sarcoma who underwent laminectomy showed no recurrence over a three-year follow-up period. This outcome may have been influenced by the adjunctive use of high-dose radiation therapy. These findings collectively underscore the importance of carefully selecting surgical approaches based on individual patient factors and tumor characteristics, as they significantly impact outcomes such as recurrence rates and overall survival in spinal tumor cases.

It is imperative to highlight that en bloc resection, despite its therapeutic potential, is associated with considerable risks, primarily due to the high morbidity rates it entails. The intricate anatomy of the spine, coupled with the proximity of essential structures, renders this surgical approach inherently hazardous. Boriani et al. [7] observed that en bloc resection, particularly in spinal surgeries, is prone to causing substantial blood loss and a heightened risk of intraoperative complications [7]. These complications frequently include injuries to adjacent organs, blood vessels, and nerves, leading to significant postoperative morbidity. Notably, their study found that nearly half of the patients experienced major complications, predominantly dural injury and vascular bleeding, with a mortality rate of 4.6% in their cohort due to severe complications. Corroborating these observations, a comprehensive review and meta-analysis by Li et al. [17], encompassing 36 studies with 961 patients, identified a complication rate of 58.3% [17]. The most common complications were neurological damage (12.7%), hardware failure (12.1%), dural tear and cerebrospinal fluid leakage (10.6%), wound-related complications (7.6%), and vascular injury and bleeding (7.3%), with an overall mortality rate of 1.2% [17]. Furthermore, Tomita et al. underscored the necessity for complex reconstructive procedures following en bloc resection, due to the removal of critical spinal structures. These reconstruction efforts are fraught with risks such as hardware failure and nonunion of the spinal segments [28]. In our study, the complication rate associated with en bloc resection was also notably high at 37.5%, with vascular bleeding and dural leakage being the primary causes. Tragically, one patient succumbed to major bleeding due to vena cava damage. These findings emphasize the crucial need for an integrated, multidisciplinary approach in managing spinal tumors. Considering the oncological prognosis is vital, and meticulous preoperative planning is essential to effectively manage potential intraoperative complications such as dural tears, vascular injuries, and significant bleeding.

The survival rates for patients undergoing en bloc or intralesional resection for spinal sarcomas remain a subject of ongoing debate. Boriani et al. [6] have advocated that en bloc resection significantly enhances survival rates in chondrosarcoma treatment, with a recurrence rate of 21% and an impressive overall survival rate of 90% Boriani et al. [6]. In contrast, they observed that intralesional resection led to a 100% local tumor recurrence rate and a markedly lower overall survival rate of 20%. Similarly, Rao et al. [23] provided congruent findings, demonstrating prolonged survival rates in patients who underwent en bloc resection compared to those who had intralesional procedures [23]a. In the case of Ewing sarcoma, however, the primary treatment approach diverges. Zhang et al. (2018) highlighted that initial decompressive surgery coupled with adjuvant chemotherapy is the preferred therapy. This combination aims to preserve or improve neurological function and ensure local tumor control. Bacci et al. [2] conducted a study focusing on the efficacy of surgical decompression in spinal Ewing sarcoma [2]. Their findings indicated that while surgical decompression was effective in symptom relief, it was the integration of chemotherapy that significantly boosted survival rates. This dual approach was pivotal in achieving local tumor control and mitigating the risk of metastatic spread. Further emphasizing the importance of chemotherapy, Bernstein et al. [3] demonstrated that the use of multi-agent chemotherapy, in conjunction with surgical decompression, led to notable improvements in survival rates [3]. Patients receiving this combination therapy exhibited a five-year survival rate significantly higher than those who underwent surgery alone. In alignment with these findings, our current study involved decompressive surgery for neurological deficits followed by adjuvant chemotherapy. This regimen facilitated local tumor control with no recurrence observed over a three-year follow-up period. Thus, the synergy of surgical decompression and chemotherapy in treating spinal Ewing sarcoma has shown promising outcomes, especially in terms of survival rates. While surgical decompression addresses the immediate physical impact of the tumor, the addition of chemotherapy is crucial for the systemic control of the disease, ultimately enhancing overall survival outcomes.

In addressing the challenges posed by spinal sarcomas, a notably rare and complex condition, our research provides crucial insights that could reshape current treatment paradigms. The scarcity of definitive guidelines for managing spinal sarcomas underscores the value of our findings. We present evidence suggesting that surgery may be a pivotal initial step in the optimal treatment strategy for these patients. This study sheds light on the potential benefits and considerable risks associated with surgical approaches, including en bloc resection and intralesional surgery, which carry risks of serious complicationsas our data indicate. Importantly, for patients with large tumors causing compression of the dural sac and neurological symptoms, surgery emerges as a potentially pivotal intervention. It can not only halt tumor progression but also preserve or enhance neurological function. Moreover, our study underscores the necessity of further research. While our retrospective analysis lays a foundation for understanding treatment needs, it is evident that prospective studies are crucial for developing more refined treatment strategies. Our ultimate goal is to extend survival and improve the quality of life for patients confronted with this formidable disease. Through continued research and clinical innovation, we aim to to provide physicians with the necessary tools and resources to effectively manage spinal sarcomas, marking a significant step forward in the field of oncological care.

## Limitations

The principal merit of this study lies in the systematic exploration of the clinical trajectory and outcomes pertaining to a range of rare tumor entities with spinal location. This research was conducted on a relatively small patient cohort, which might initially appear as a limitation. However, given that extant data on these diseases predominantly emerge from case reports, we posit that our findings offer a comprehensive and pragmatic portrayal of these afflictions. It is important to acknowledge potential selection bias, which may have been introduced due to the retrospective design of the study. Additionally, the limited case numbers precluded the possibility of conducting a multivariate analysis for the loss of ambulation. A significant limitation of this retrospective study is the difficulty in achieving consistent long-term follow-up. Due to the nature of retrospective research, our follow-up data was restricted to existing medical records and personal communications, which often resulted in incomplete datasets. Additionally, the variation in follow-up duration among patients, coupled with some being lost to follow-up over time, poses challenges in accurately assessing long-term outcomes such as disease recurrence and survival rates. These factors are inherent to the study design and reflect the practical difficulties encountered in gathering comprehensive follow-up data in a retrospective framework. Another limitation of this study is the small number of spinal sarcoma cases treated at our center, which may impact the generalizability of our findings. The infrequent nature of these cases limits our ability to perform extensive statistical analysis or to compare different treatment modalities extensively. This small sample size is a common challenge in studies involving rare diseases and should be considered when interpreting our results and conclusions.

## Conclusions

Sarcomas affecting the spine, whether primary or metastatic, represent a rare category of malignancies, characterized by limited treatment options. Surgical intervention stands as a crucial therapeutic strategy, offering not only palliative relief but also functional benefits, and contribute to extend the patient's survival. The consideration of adjuvant therapy, tailored according to the specific type of tumor, is essential. A multidisciplinary approach is of paramount importance in determining the most effective treatment plan for these complex cases, ensuring a comprehensive and patient-centric therapy strategy.

## Data Availability

The datasets generated and/or analyzed during the current study are available from the corresponding author on reasonable request.

## Code availability

The code supporting the findings of this study is available from the corresponding author upon reasonable request.
